# Supplementary material for: In silico assessment of primers for eDNA studies using PrimerTree and application to characterize the biodiversity surrounding the Cuyahoga River
Source: Sci Rep. 2016 Mar 11;6:22908. doi: 10.1038/srep22908 (PMC4786790; doi:10.1038/srep22908)
Supplement: Supplementary Information [file srep22908-s1.pdf]

Supplemental Table and Figure Legends for

"In silico assessment of primers for eDNA studies using PrimerTree and application to characterize the biodiversity surrounding the Cuyahoga River"

Cannon MV, Hester J, Shalkhauser A, Chan ER, Logue K, Small ST and Serre D

Supplemental Table 1: Sample descriptions

Supplemental Table 2: Primer sequences

Supplemental Table 3: Primer length and specificity based on observed and *in silico* data (in brackets)

Supplemental Table 4: Taxonomy and raw read counts for vascular plant and bryophyte primers

Supplemental Table 5: Fish species identified using the mammalian 16S rRNA and fish mt-Cytb primers

Supplemental Table 6: Raw read counts and species absence/presence calls for macroorganisms

Supplemental Table 7: Experimental expense summary

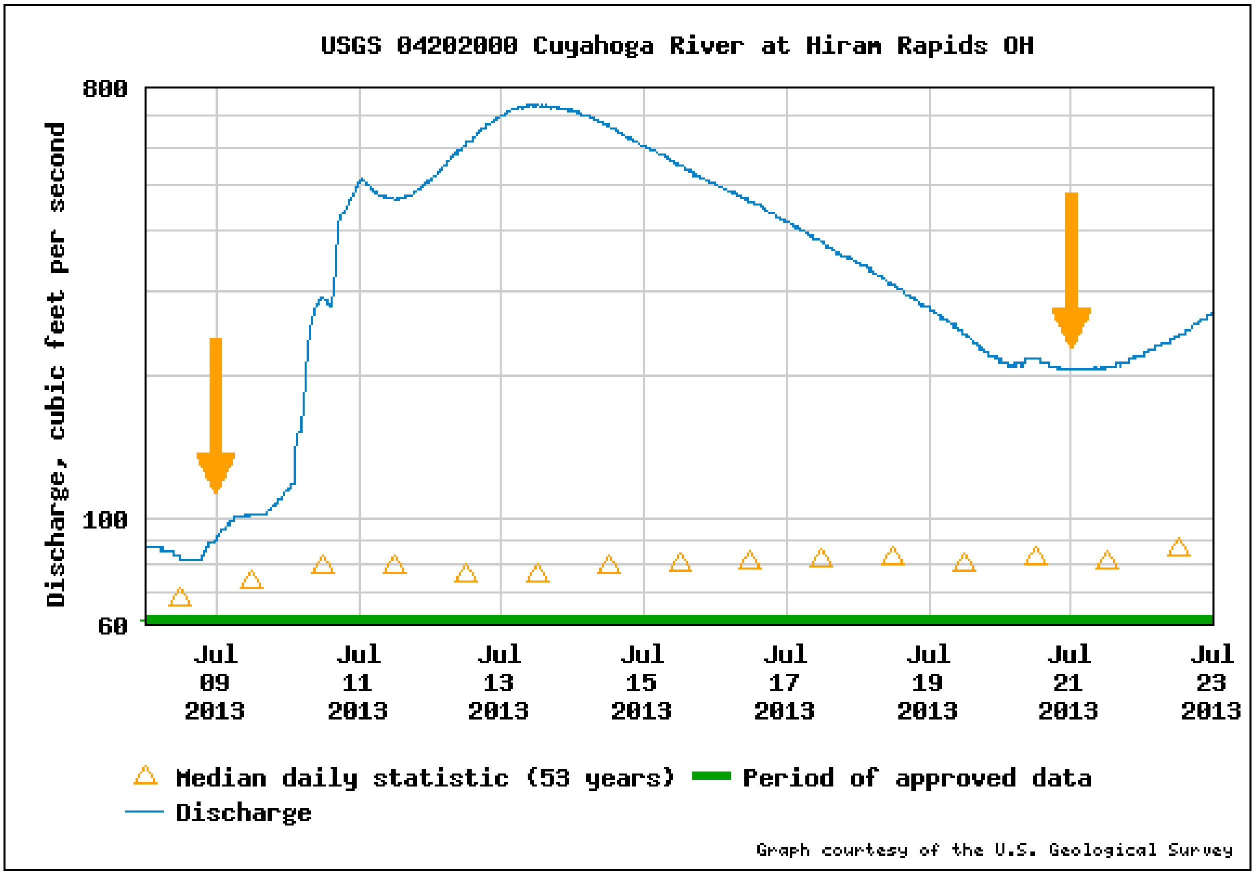

Supplemental Figure 1: USGS discharge at the days of sample collection. The figure shows the discharge of the Cuyahoga River at Hiram Rapids (Upper Cuyahoga, OH) in cubic feet per second (y-axis, in log scale) between July 9 and July 24 2013. The days of collection are indicated by the orange arrows.

Supplemental Figure 2: PrimerTree results for the 12 primer pairs. Each primer set is denoted at the top of the page. Each page is divided into eight sections that show different taxonomic levels, with the points colored according to the taxa within that level. Levels with few taxa have colored text labels. The guide below each tree shows the scale in number of nucleotide differences.

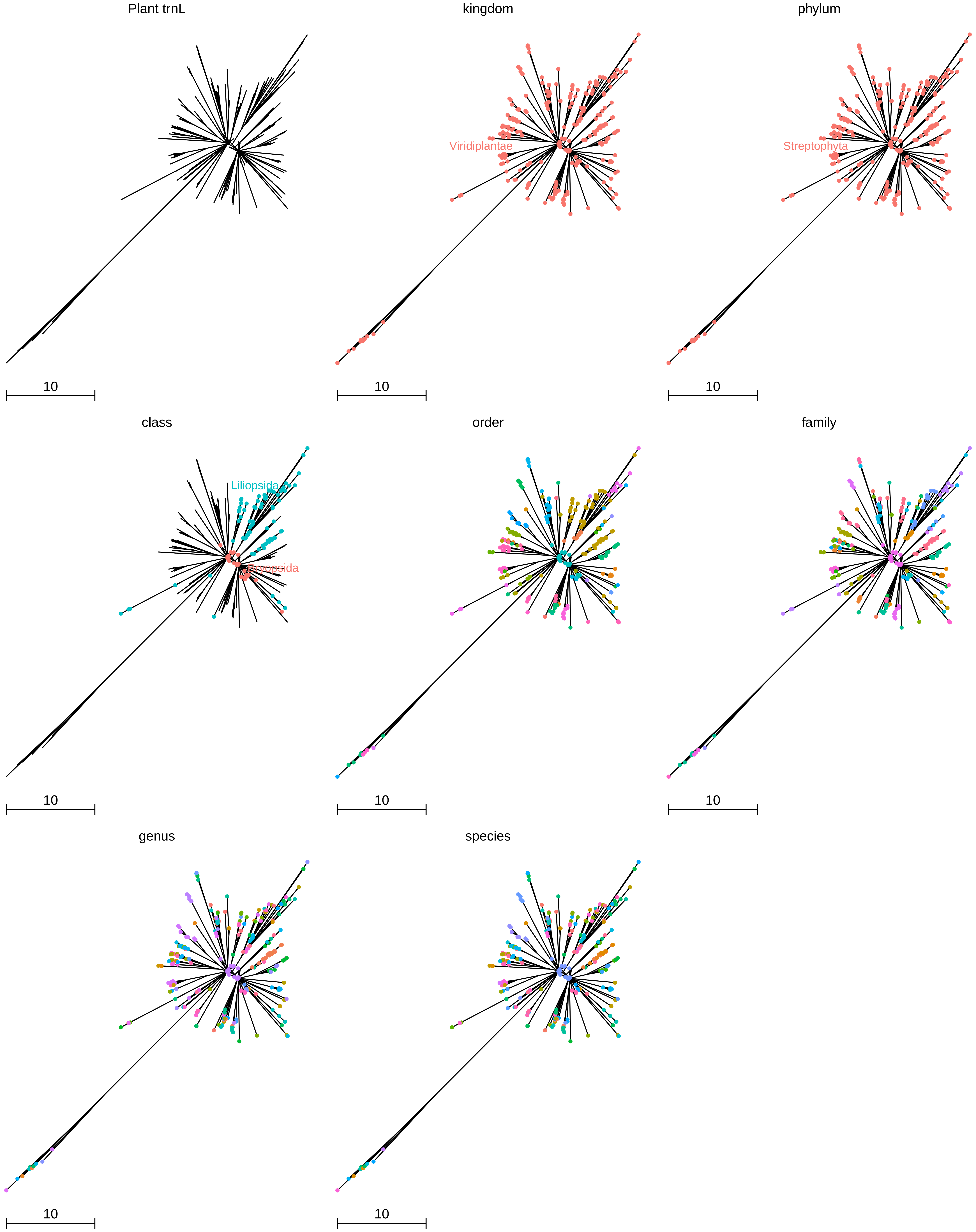
Plant trnL
10
kingdom
Viridiplantae
10
phylum
Streptophyta
10
class
Liliopsida
Bryopsida
10
order
10
family
10
genus
10
species
10

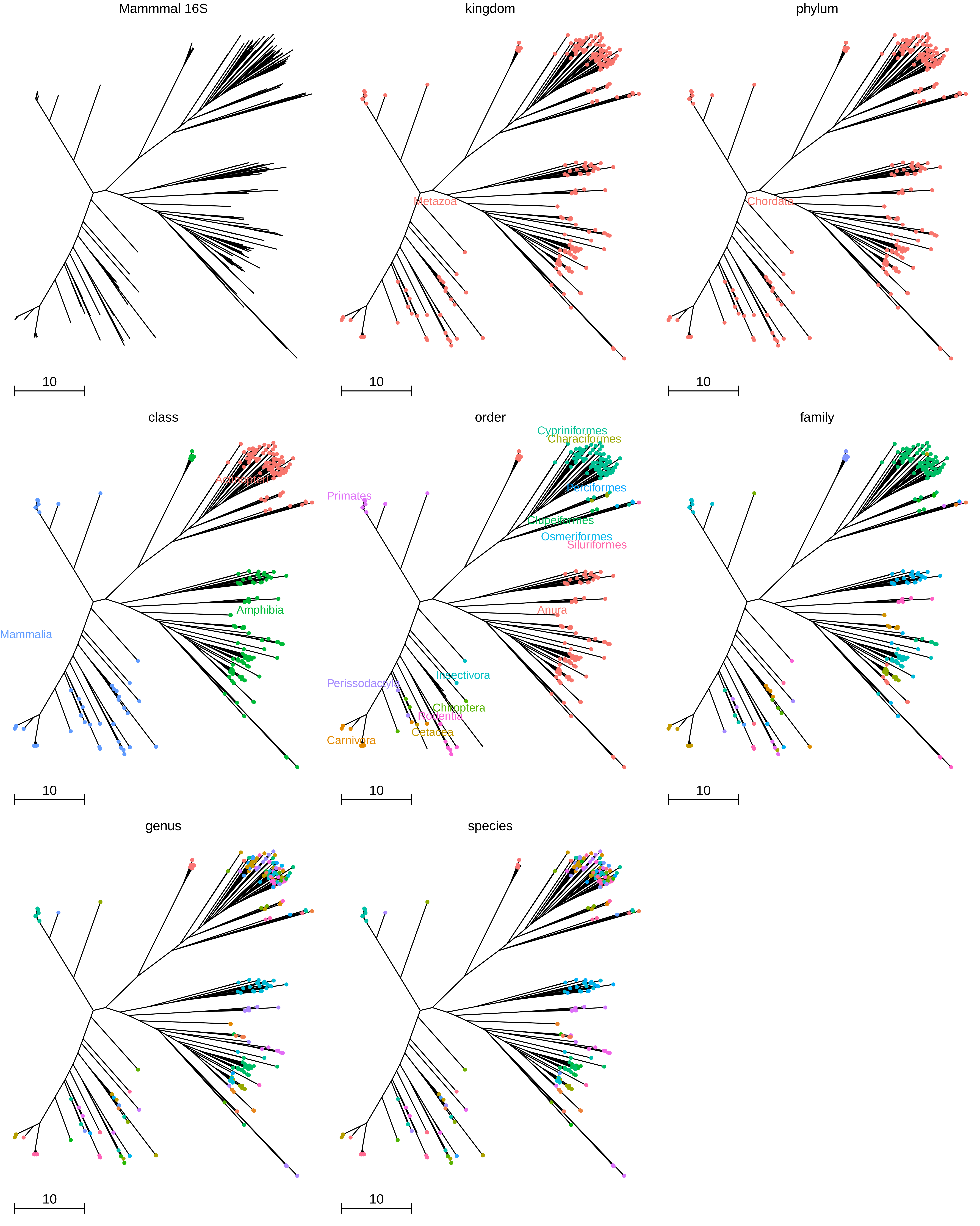

Mammmal 16S
kingdom
phylum
Metazoa
Chordata
10
class
order
family
Actinopteri
Amphibia
Mammalia
Cypriniformes
Characiformes
Perciformes
Clupeiformes
Osmeriformes
Siluriformes
Primates
Anura
Insectivora
Perissodactyla
Chiroptera
Rodentia
Cetacea
Carnivora
genus
species

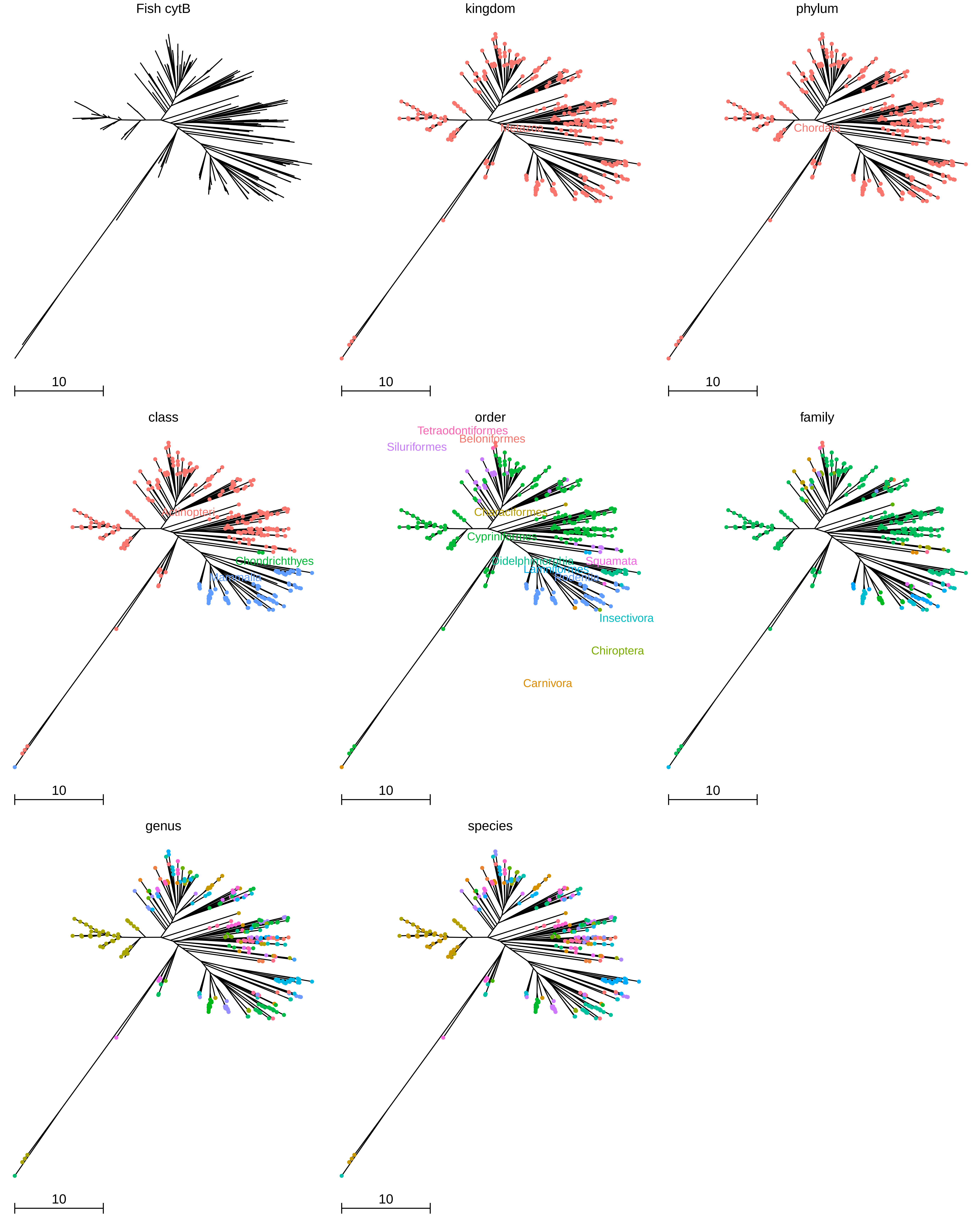
Fish cytB
10
kingdom
Metazoa
10
phylum
Chordata
10
class
Actinopteri
Chondrichthyes
Mammalia
10
order
Tetraodontiformes
Beloniformes
Siluriformes
Characiformes
Cypriniformes
Didelphimorphia
Lamniformes
Squamata
Rodentia
Insectivora
Chiroptera
Carnivora
10
family
10
genus
10
species
10

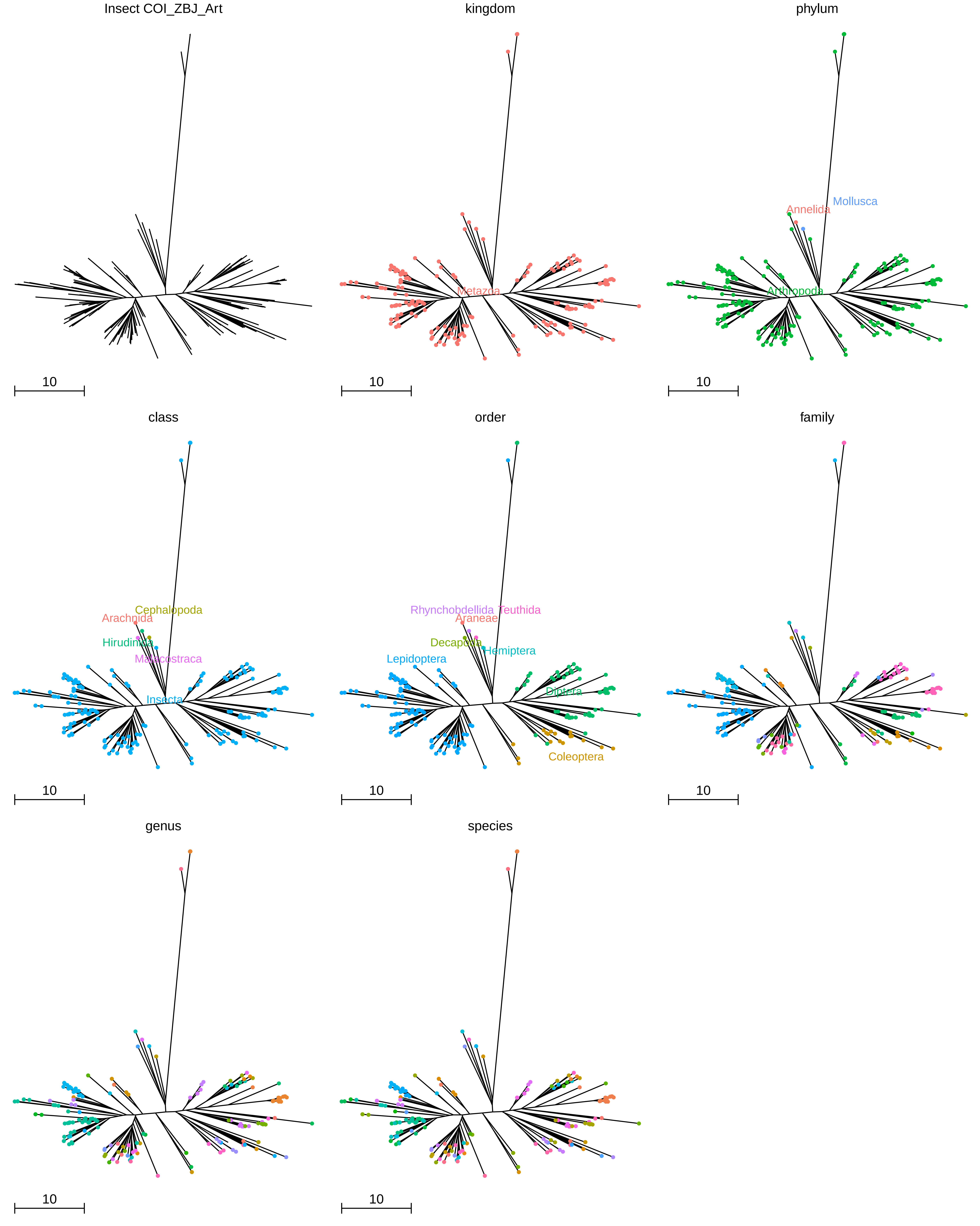

Insect COI_ZBJ_Art
kingdom
phylum
class
order
family
genus
species
Metazoa
Annelida
Mollusca
Arthropoda
Cephalopoda
Arachnida
Hirudinida
Malacostraca
Insecta
Rhynchobdellida
Teuthida
Araneae
Decapoda
Hemiptera
Lepidoptera
Diptera
Coleoptera
10

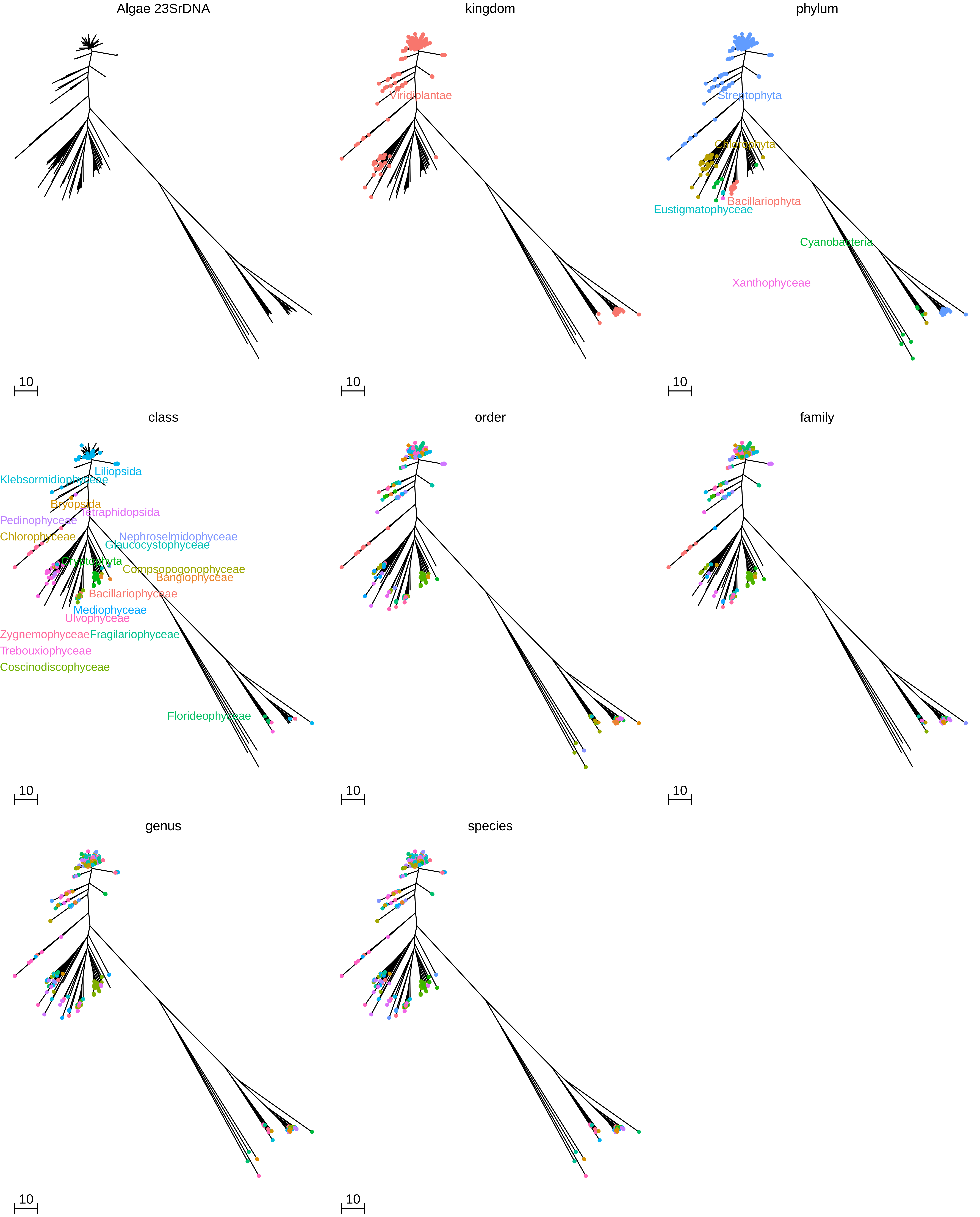

Algae 23SrDNA
kingdom
phylum
class
order
family
genus
species
Viridiplantae
Streptophyta
Chlorophyta
Bacillariophyta
Eustigmatophyceae
Cyanobacteria
Xanthophyceae
Liliopsida
Klebsormidiophyceae
Bryopsida
Tetraphidopsida
Pedinophyceae
Chlorophyceae
Nephroselmidophyceae
Glaucocystophyceae
Cryptophyta
Compsopogonophyceae
Bangiophyceae
Bacillariophyceae
Mediophyceae
Ulvophyceae
Zygnemophyceae
Fragilariophyceae
Trebouxiophyceae
Coscinodiscophyceae
Florideophyceae
10

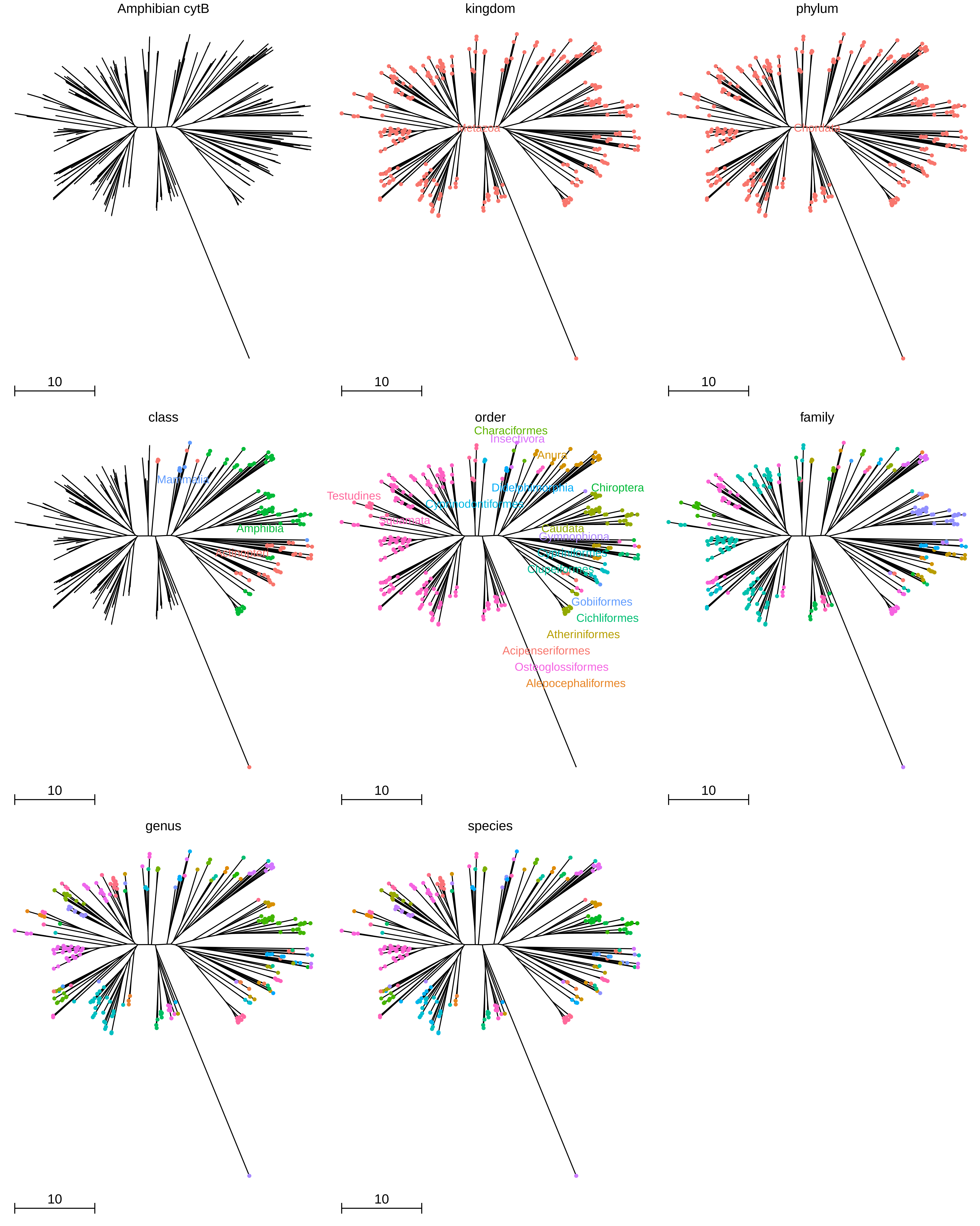
Amphibian cytB
10
kingdom
Metazoa
10
phylum
Chordata
10
class
Mammalia
Amphibia
Actinopteri
10
order
Characiformes
Insectivora
Anura
Testudines
Didelphimorphia
Chiroptera
Cyprinodontiformes
Squamata
Caudata
Gymnophiona
Cypriniformes
Clupeiformes
Gobiiformes
Cichliformes
Atheriniformes
Acipenseriformes
Osteoglossiformes
Alepocephaliformes
10
family
10
genus
10
species
10

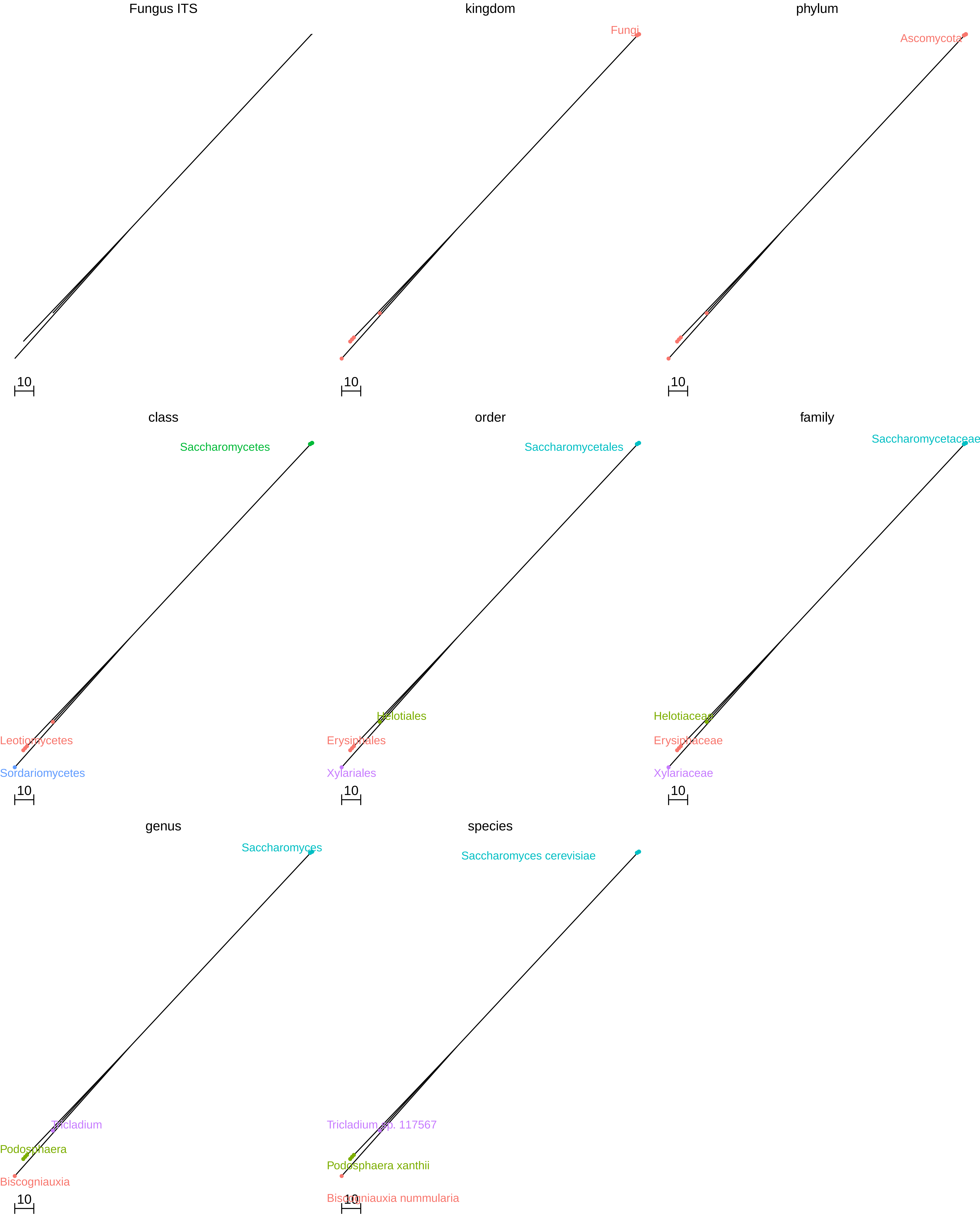

Fungus ITS
10
kingdom
Fungi
10
phylum
Ascomycota
10
class
Saccharomycetes
Leotiomycetes
Sordariomycetes
10
order
Saccharomycetales
Helotiales
Erysiphales
Xylariales
10
family
Saccharomycetaceae
Helotiaceae
Erysiphaceae
Xylariaceae
10
genus
Saccharomyces
Tricladium
Podosphaera
Biscogniauxia
10
species
Saccharomyces cerevisiae
Tricladium sp. 117567
Podosphaera xanthii
Biscogniauxia nummularia
10

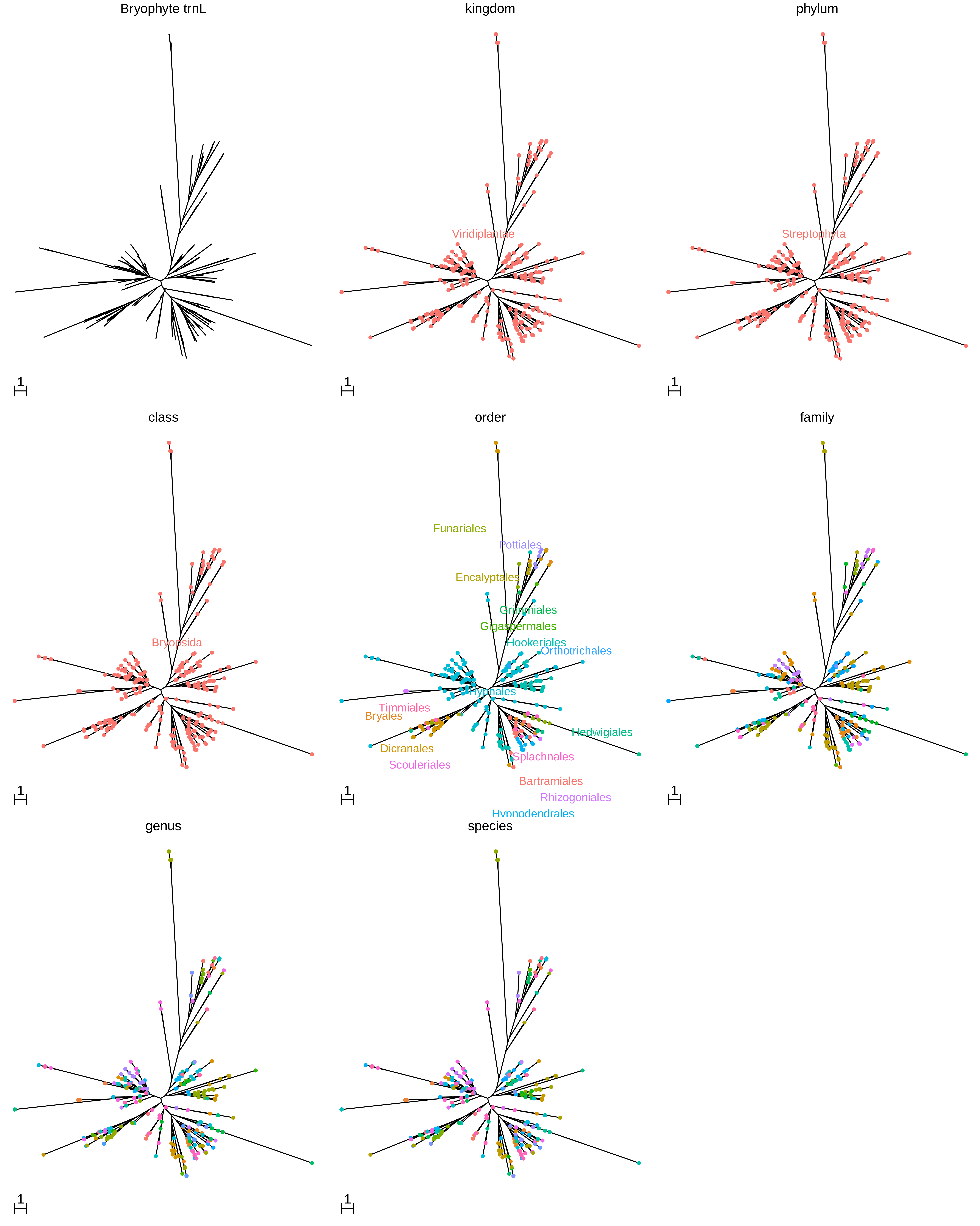
Bryophyte trnL
kingdom
phylum
Viridiplantae
Streptophyta
class
order
family
Bryopsida
Funariales
Pottiales
Encalyptales
Grimmiales
Gigaspermales
Hookeriales
Orthotrichales
Hypnales
Timmiales
Bryales
Hedwigiales
Dicranales
Splachnales
Scouleriales
Bartramiales
Rhizogoniales
Hypnodendrales
genus
species
1

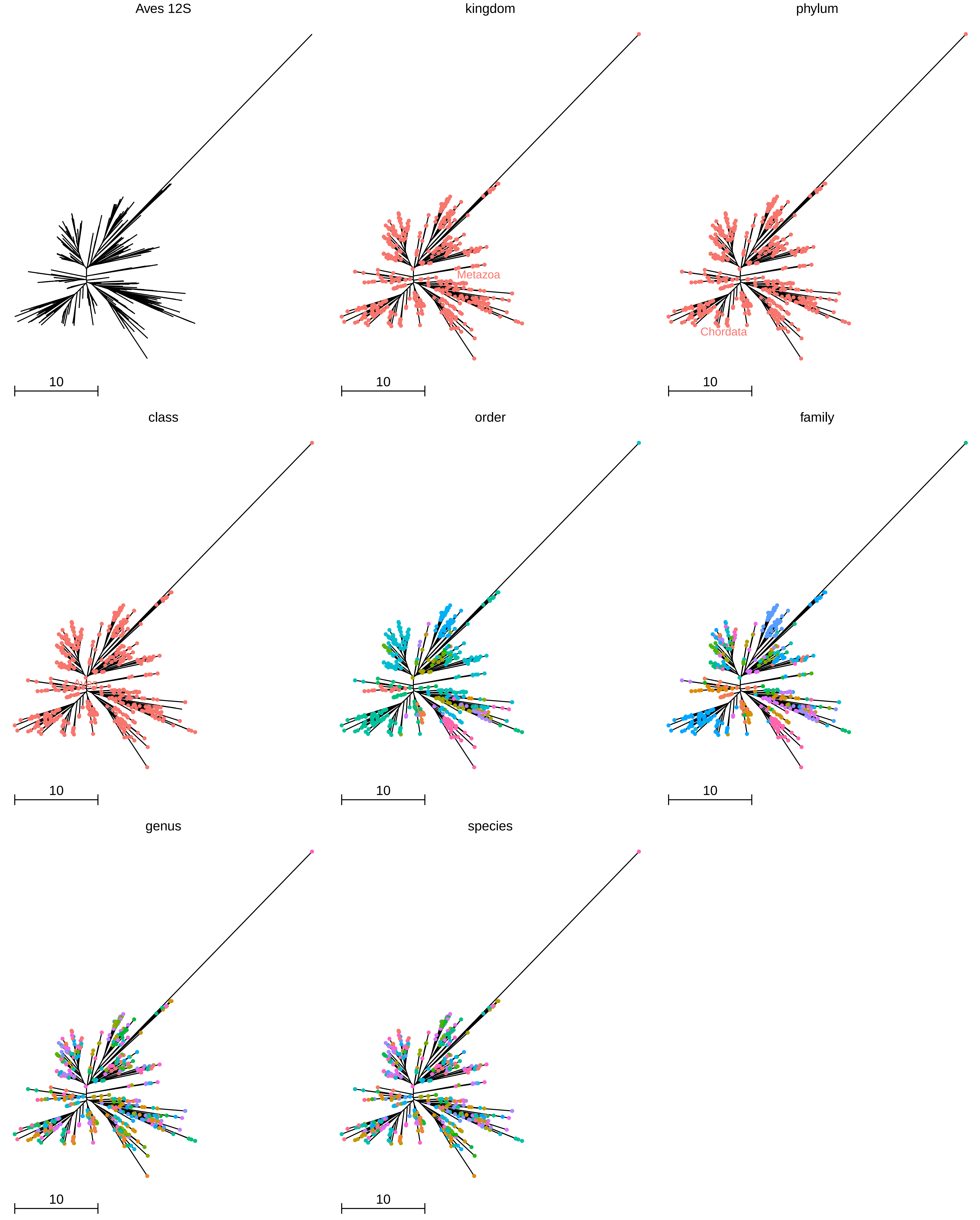
Aves 12S
10
kingdom
Metazoa
10
phylum
Chordata
10
class
Aves
10
order
10
family
10
genus
10
species
10

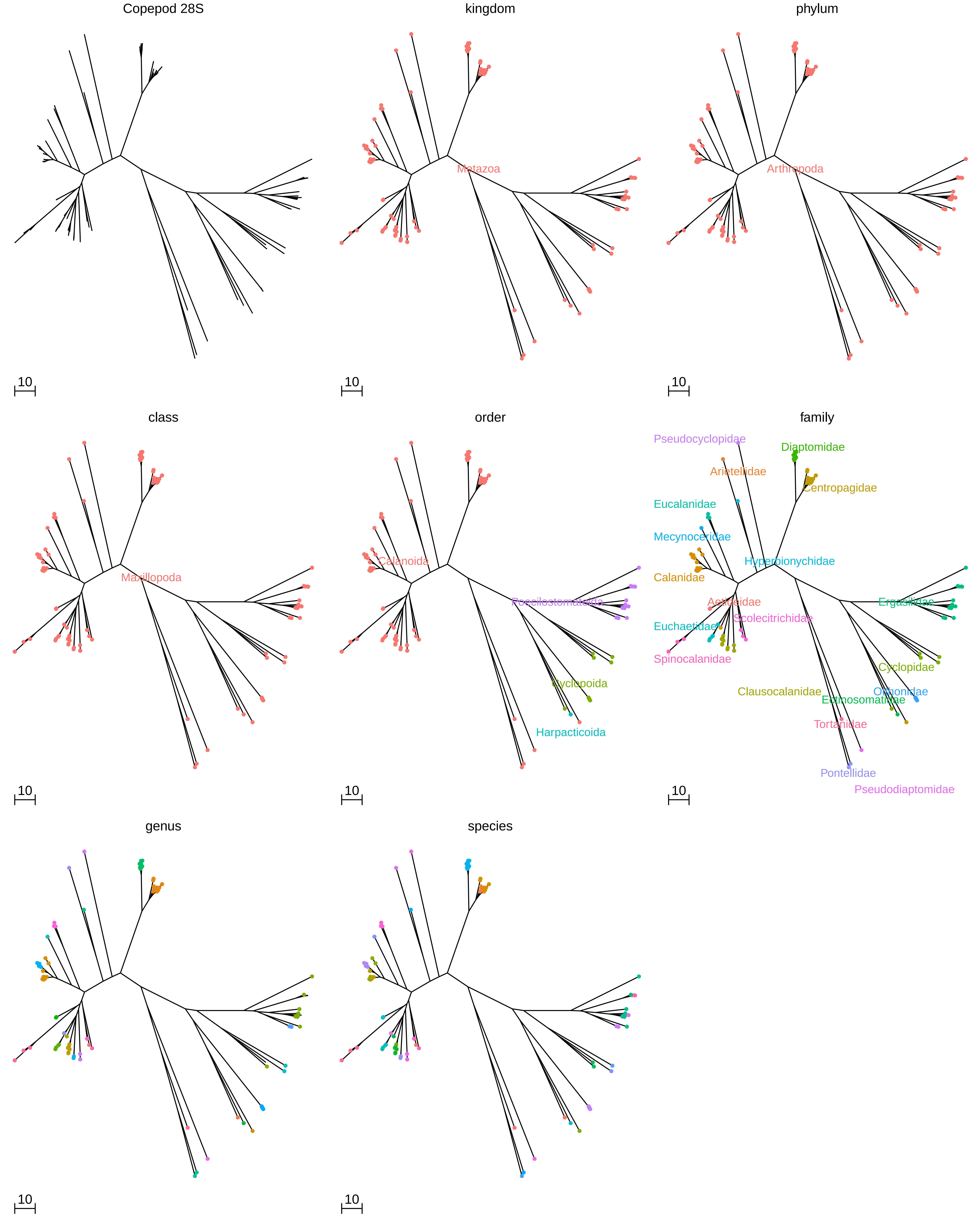

Copepod 28S
kingdom
phylum
class
order
family
genus
species
10
Metazoa
Arthropoda
Maxillopoda
Calanoida
Poecilostomatoida
Cyclopoida
Harpacticoida
Pseudocyclopidae
Diaptomidae
Arietellidae
Centropagidae
Eucalanidae
Mecynoceridae
Hyperbionychidae
Calanidae
Aetideidae
Scolecitrichidae
Ergasilidae
Euchaetidae
Spinocalanidae
Cyclopidae
Clausocalanidae
Ectinosomatidae
Oithonidae
Tortanidae
Pontellidae
Pseudodiaptomidae

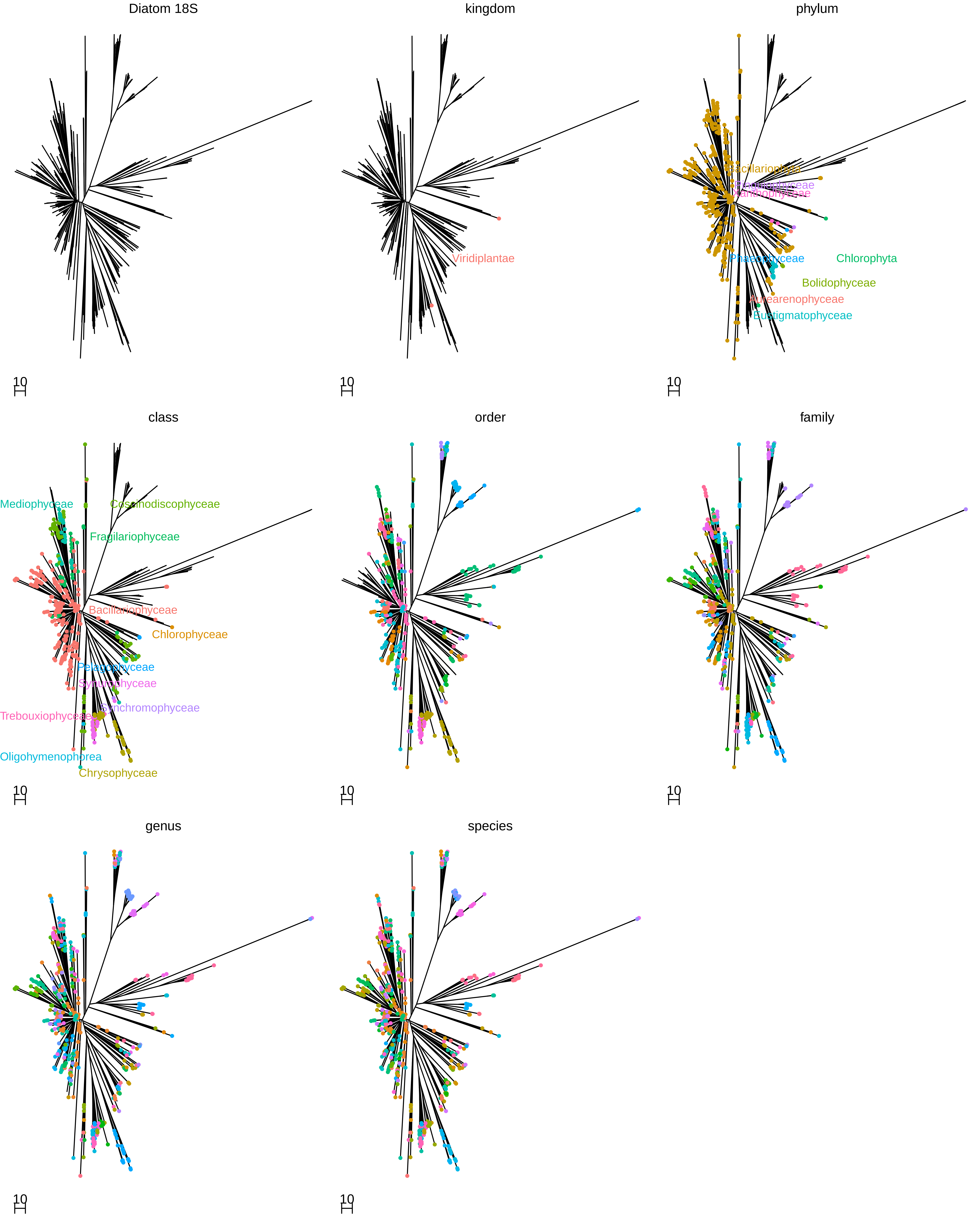
Diatom 18S
kingdom
phylum
Viridiplantae
Bacillariophyta
Xanthophyceae
Phaeophyceae
Chlorophyta
Bolidophyceae
Aurearenophyceae
Eustigmatophyceae
class
order
family
Mediophyceae
Coscinodiscophyceae
Fragilariophyceae
Bacillariophyceae
Chlorophyceae
Pelagophyceae
Synurophyceae
Synchromophyceae
Trebouxiophyceae
Oligohymenophorea
Chrysophyceae
genus
species
10

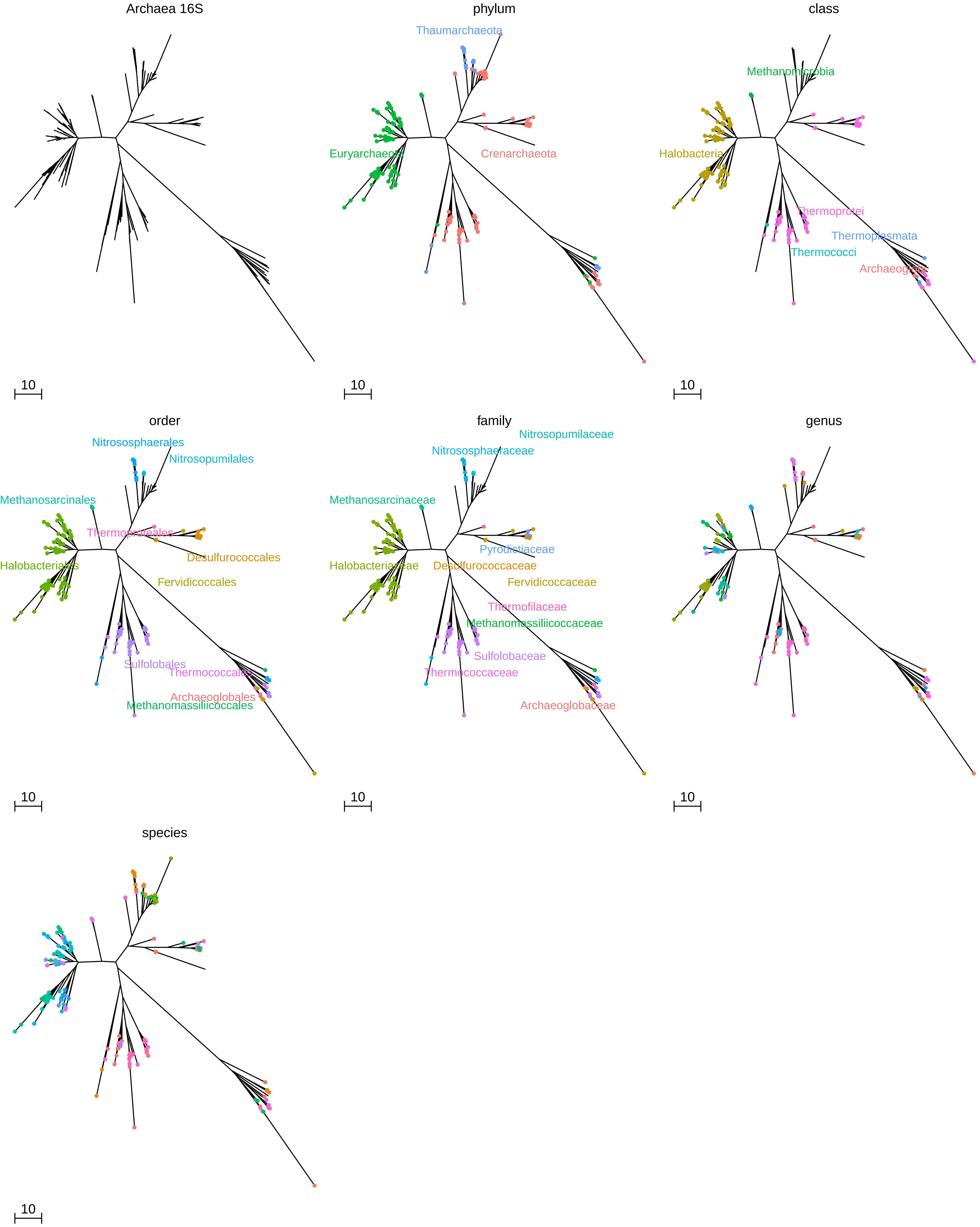
Archaea 16S
10
phylum
Thaumarchaeota
Euryarchaeota
Crenarchaeota
10
class
Methanomicrobia
Halobacteria
Thermoprotei
Thermoplasmata
Thermococci
Archaeoglobi
10
order
Nitrososphaerales
Nitrosopumilales
Methanosarcinales
Thermoproteales
Desulfurococcales
Halobacteriales
Fervidicoccales
Sulfolobales
Thermococcales
Archaeoglobales
Methanomassiliicoccales
10
family
Nitrosopumilaceae
Nitrososphaeraceae
Methanosarcinaceae
Pyrodictiaceae
Halobacteriaceae
Desulfurococcaceae
Fervidicoccaceae
Thermofilaceae
Methanomassiliicoccaceae
Sulfolobaceae
Thermococcaceae
Archaeoglobaceae
10
genus
10
species
10

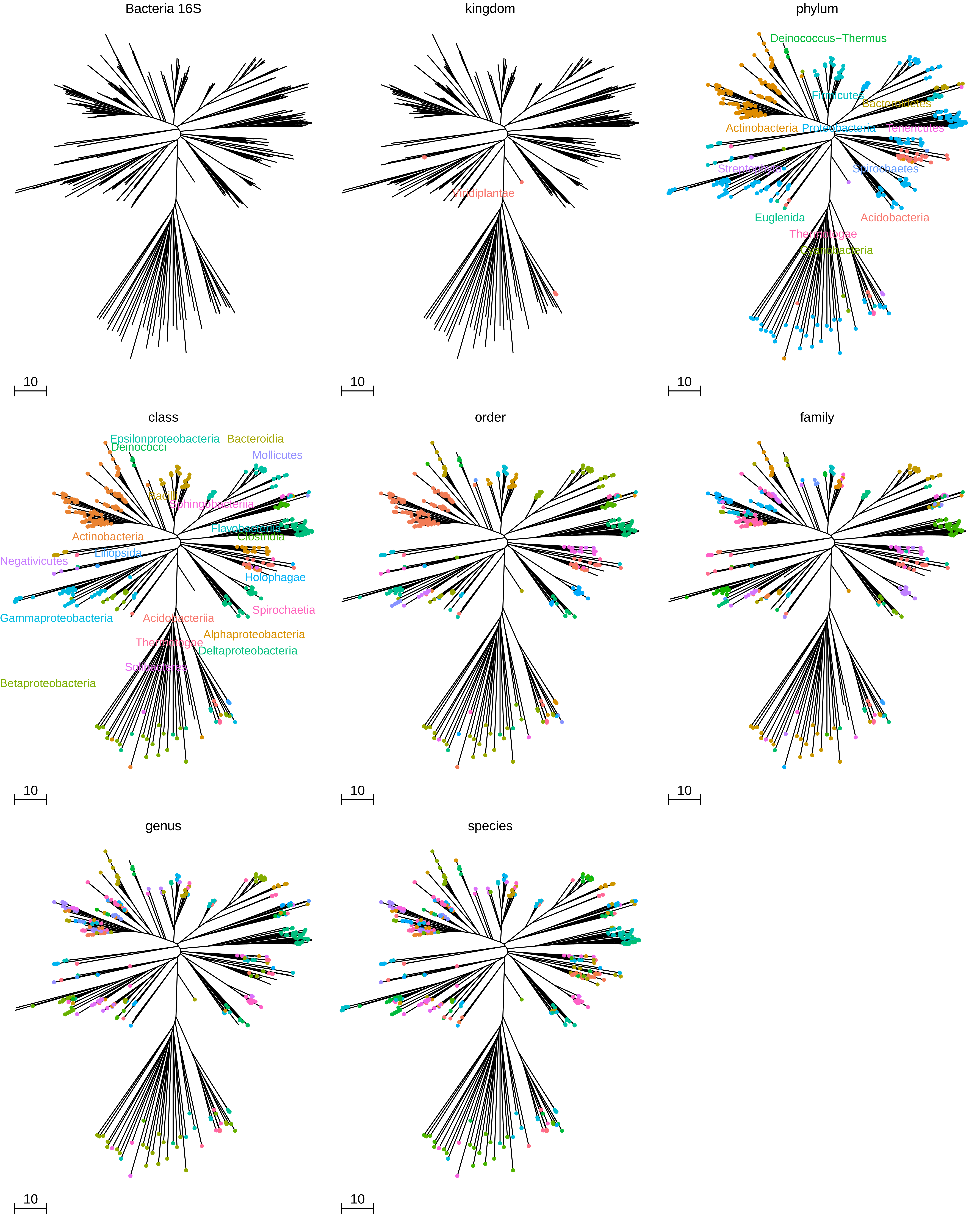

Bacteria 16S
kingdom
phylum
Deinococcus−Thermus
Firmicutes
Bacteroidetes
Actinobacteria
Proteobacteria
Tenericutes
Streptophyta
Spirochaetes
Euglenida
Acidobacteria
Thermotogae
Cyanobacteria
Viridiplantae
class
Epsilonproteobacteria
Deinococci
Bacteroidia
Mollicutes
Bacilli
Sphingobacteriia
Flavobacteriia
Clostridia
Actinobacteria
Liliopsida
Negativicutes
Holophagae
Gammaproteobacteria
Acidobacteriia
Spirochaetia
Alphaproteobacteria
Thermotogae
Deltaproteobacteria
Solibacteres
Betaproteobacteria
order
family
genus
species
10

Supplemental Table 1

ID	Day	Sample	LAT	LON	Notes	Time	Portion	Rain	InRiver
1	1	DS92	41.38616793	-81.15735861		8:11 AM	Upper	Before	yes
2	1	DS24	41.38289094	-81.15737203		8:26 AM	Upper	Before	yes
3	1	DS80	41.38242415	-81.15462989		8:36 AM	Upper	Before	yes
4	1	DS39	41.37719879	-81.15318334		8:50 AM	Upper	Before	yes
5	1	DS85	41.37009471	-81.16215114		9:05 AM	Upper	Before	yes
6	1	DS28	41.3629835	-81.16263611		9:22 AM	Upper	Before	yes
7	1	DS1	41.34894742	-81.16448415		9:39 AM	Upper	Before	yes
8	1	DS63	41.34008037	-81.16737968		9:50 AM	Upper	Before	yes
9	1	DS66	41.32691433	-81.17250907		10:13 AM	Upper	Before	yes
10	1	DS87	41.31662386	-81.18341435		10:38 AM	Upper	Before	yes
11	1	DS74	41.31437793	-81.19217578	Brook coming out of the culvert	10:51 AM	Upper	Before	no
12	1	DS86	41.31015153	-81.1946922		11:02 AM	Upper	Before	yes
13	1	DS23	41.30765162	-81.1960903	Water from pipe	11:08 AM	Upper	Before	no
14	1	DS61	41.30293823	-81.20072516		11:24 AM	Upper	Before	no
15	1	DS22	41.30293823	-81.20072516			Upper	Before	yes
16	1	DS5	41.30293823	-81.20072516	Pond next to river	11:26 AM	Upper	Before	no
17	1	DS38	41.2907584	-81.21081403		11:43 AM	Upper	Before	yes
18	1	DS25	41.28487037	-81.21658481	Retention spill - outside/river	11:56 AM	Upper	Before	yes
19	1	DS69	41.28487037	-81.21658481	Retention spill - inside		Upper	Before	yes
21	1	DS81	41.28268839	-81.21983078	Drainage pipe	12:03 PM	Upper	Before	no
22	1	DS88	ND	ND	100 m downstream of 21	12:05 PM	Upper	Before	yes
23	1	DS32	41.2797217	-81.22239229		12:12 PM	Upper	Before	yes
24	1	DS58	41.27846492	-81.22087961	Brook	12:18 PM	Upper	Before	no
25	1	DS89	ND	ND	WWTP Mantua	12:27 PM	Upper	Before	yes
26	1	DS15	41.27651059	-81.22707878		12:31 PM	Upper	Before	yes
27	1	DS10	41.27373467	-81.23310386		12:40 PM	Upper	Before	yes
28	1	DS52	41.26907567	-81.24587134		12:58 PM	Upper	Before	yes
29	1	DS75	41.25905394	-81.25497258		1:13 PM	Upper	Before	yes
30	1	DS94	41.25213861	-81.26298711		1:29 PM	Upper	Before	yes
31	1	DS90	41.24900957	-81.28321608		1:47 PM	Upper	Before	yes
32	1	DS72	41.12303434	-81.51505256		3:36 PM	Middle	Before	yes
33	1	DS83	41.12168653	-81.51962757		4:03 PM	Middle	Before	yes
34	1	DS6	41.11745937	-81.52206712		4:25 PM	Middle	Before	yes
35	1	DS67	41.11906174	-81.52647978		4:40 PM	Middle	Before	yes
36	1	DS27	41.12984237	-81.5381261		4:57 PM	Middle	Before	yes
37	1	DS36	41.13340736	-81.54780149	Double-drain	5:10 PM	Middle	Before	yes
38	1	DS37	41.13783586	-81.55639571		5:21 PM	Middle	Before	yes
39	1	DS62	ND	ND	100 m downstream 38, near drain	5:25 PM	Middle	Before	yes
40	1	DS13	41.14409513	-81.56314046		5:38 PM	Middle	Before	yes
41	1	DS91	41.15556702	-81.57106698	Sewer plant	5:49 PM	Middle	Before	yes
42	1	DS53	41.15980189	-81.57297227	WPCS Akron Waterfall	5:56 PM	Middle	Before	no
43	1	DS55	ND	ND	Flooded river in morning		Middle	After	yes
1	2	DS77	41.123059	-81.515063		10:34 AM	Middle	After	yes
2	2	DS50	41.117976	-81.524711		11:43 AM	Middle	After	yes
3	2	DS47	41.136665	-81.547141		11:59 AM	Middle	After	yes
5	2	DS18	41.181709	-81.58388		12:18 PM	Middle	After	yes
6	2	DS49	41.241662	-81.548578			Middle	After	yes
7	2	DS65	41.262888	-81.560156		12:49 PM	Middle	After	yes
10	2	DS46	41.392993	-81.629411			Lower	After	yes
12	2	DS51	41.414974	-81.638878		2:00 PM	Lower	After	yes
13	2	DS64	41.416104	-81.639279		2:06 PM	Lower	After	yes
14	2	DS14	41.417085	-81.645042	From covered drain	2:13 PM	Lower	After	no
15	2	DS4	41.419943	-81.654479	From large affluent of clear water from WWTP	2:19 PM	Lower	After	no
16	2	DS60	41.420158	-81.657798		2:26 PM	Lower	After	yes
17	2	DS84	41.422185	-81.663617	Drain LB	2:34 PM	Lower	After	no
18	2	DS26	41.42701	-81.666138	LB before suspended pipelines	2:42 PM	Lower	After	yes
19	2	DS21	41.434384	-81.664195	Near Sunoco Refinery	2:52 PM	Lower	After	yes
20	2	DS57	41.439323	-81.669733		3:03 PM	Lower	After	yes
21	2	DS41	41.443194	-81.677473		3:09 PM	Lower	After	yes

22_out	2	DS7	41.444157	-81.677299	Pipe + outside retention line	3:13 PM	Lower	After	yes
22_in	2	DS34	41.444157	-81.677299	Pipe + inside retention line		Lower	After	yes
23	2	DS54	41.448046	-81.684159		3:25 PM	Lower	After	yes
24	2	DS2	41.449179	-81.681288	ArcelorMittal Outfall	3:28 PM	Lower	After	no
25	2	DS35	41.460835	-81.683129		3:47 PM	Lower	After	yes
26	2	DS19	41.464189	-81.679458	Drain from metal basin	3:52 PM	Lower	After	no
27	2	DS40	41.467604	-81.671395		4:04 PM	Lower	After	yes
28	2	DS70	41.475447	-81.670943		4:23 PM	Lower	After	yes
29	2	DS9	41.481574	-81.675583		4:32 PM	Lower	After	yes
30	2	DS3	41.485641	-81.678992	Shelly Liquid terminal	4:42 PM	Lower	After	yes
31	2	DS45	41.489249	-81.684129		4:50 PM	Lower	After	yes
32	2	DS71	41.487623	-81.693352		5:06 PM	Lower	After	yes
33	2	DS78	41.493653	-81.692506		5:13 PM	Lower	After	yes
34	2	DS12	41.488853	-81.703449		5:31 PM	Lower	After	yes
35	2	DS95	41.495575	-81.702833		5:43 PM	Lower	After	yes
36	2	DS43	41.500043	-81.708604		5:59 PM	Lower	After	yes
37	2	DS82	41.503674	-81.712785	In harbor, end of pier before Wendy Park	6:02 PM	Lower	After	yes
1	M	DS33	41.4252	-81.15651667			Upper	After	yes
2	M	DS20	41.41858333	-81.15888333			Upper	After	yes
3	M	DS17	41.4125	-81.15595			Upper	After	yes
4	M	DS68	41.40428333	-81.16113333			Upper	After	yes
5	M	DS11	41.39693333	-81.15885			Upper	After	yes
6	M	DS79	41.3908	-81.15976667			Upper	After	yes
7	M	DS31	41.38608333	-81.15741667			Upper	After	yes
8	M	DS42	41.38158333	-81.1584			Upper	After	yes
9	M	DS30	41.37728333	-81.15076667			Upper	After	yes
10	M	DS76	41.37473333	-81.1563			Upper	After	yes
11	M	DS73	41.36871667	-81.15995			Upper	After	yes
12	M	DS93	41.3609	-81.1626			Upper	After	yes
13	M	DS59	41.35051667	-81.1637			Upper	After	yes
14	M	DS8	41.33803333	-81.16733333			Upper	After	yes
15	M	DS48	41.32778333	-81.17135			Upper	After	yes

Supplemental Table 2

Primer	Forward Primer	Reverse Primer	Target	Citation
trnL	CTTTCCCTACACGACGCTCTTCCGATCTGGGCAATCCTGAGCCAA	GTGACTGGAGTTCAGACGTGTGCTCTTCCGATCTCCATTGAGTCTCTGCACCTATC	Vascular plants	Taberlet et al. 2007
16Smam	CTTTCCCTACACGACGCTCTTCCGATCTCGGTTGGGGTGACCTCGGA	GTGACTGGAGTTCAGACGTGTGCTCTTCCGATCTGCTGTTATCCCTAGGGTAACT	Mammals	Taylor, 1996
COI_ZBJ_Art	CTTTCCCTACACGACGCTCTTCCGATCTAGATATTGGAACWTTATATTTTATTTTTGG	GTGACTGGAGTTCAGACGTGTGCTCTTCCGATCTWACTAATCAATTWCCAAATCCTCC	Insects+Arachn	Zeale et al., 2011
23SrDNA	CTTTCCCTACACGACGCTCTTCCGATCTGGACAGAAAGACCCTATGAA	GTGACTGGAGTTCAGACGTGTGCTCTTCCGATCTTGAGTGACGGCCTTTCCACT	Algae	Sherwood and Presting, 2007
AmpCB	CTTTCCCTACACGACGCTCTTCCGATCTAGTCCTGTTGGGTTGGTTGACCCNGTTT	GTGACTGGAGTTCAGACGTGTGCTCTTCCGATCTAATGCAACTCTCACCCGATTCTT	Amphibians	Thomsen et al., 2012
FungusITS	CTTTCCCTACACGACGCTCTTCCGATCTGGAAGTAAAAGTCGTAACAAGG	GTGACTGGAGTTCAGACGTGTGCTCTTCCGATCTCAAGAGATCCGTTGTTGAAAGTT	Fungi	Epp et al., 2012
BryoTrnL	CTTTCCCTACACGACGCTCTTCCGATCTGATTCAGGGAAACTTAGGTTG	GTGACTGGAGTTCAGACGTGTGCTCTTCCGATCTCCATTGAGTCTCTGCACC	Bryophytes	Epp et al., 2012
Aves12S	CTTTCCCTACACGACGCTCTTCCGATCTGATTAGATACCCCACTATGC	GTGACTGGAGTTCAGACGTGTGCTCTTCCGATCTGTTTTAAGCGTTTGTGCTCG	Birds	Epp et al., 2012
Cop28S	CTTTCCCTACACGACGCTCTTCCGATCTTGTGTGGTGGTAAACGGAG	GTGACTGGAGTTCAGACGTGTGCTCTTCCGATCTCCGCCGACCTACTCG	Copepod	Bisset et al., 2005
Diatom18S	CTTTCCCTACACGACGCTCTTCCGATCTTCCAGCTCCAATAGCG	GTGACTGGAGTTCAGACGTGTGCTCTTCCGATCTCTACGATGGTATCTAATC	Diatom	Zimmerman et al., 2011
FishCB	CTTTCCCTACACGACGCTCTTCCGATCTTCCTTTTGAGGCGCTACAGT	GTGACTGGAGTTCAGACGTGTGCTCTTCCGATCTGGAATGCGAAGAATCGTGTT	Fish	Thomsen et al., 2012
Archaea16S	CTTTCCCTACACGACGCTCTTCCGATCTCCGACGGTGAGRGRYGAA	GTGACTGGAGTTCAGACGTGTGCTCTTCCGATCTGTGCTCCCCCGCCAATTCCT	Archaea	Baker et al., 2003
**Barcoding Primer**	**Primer**	**Barcode**		
DS01	CAAGCAGAAGACGGCATACGAGATAACCGCGTGACTGGAGTTC	AACCGC		
DS02	CAAGCAGAAGACGGCATACGAGATAACGCCGTGACTGGAGTTC	AACGCC		
DS03	CAAGCAGAAGACGGCATACGAGATAAGCGGGTGACTGGAGTTC	AAGCGG		
DS04	CAAGCAGAAGACGGCATACGAGATAAGGCGGTGACTGGAGTTC	AAGGCG		
DS05	CAAGCAGAAGACGGCATACGAGATACACAGGTGACTGGAGTTC	ACACAG		
DS06	CAAGCAGAAGACGGCATACGAGATACACTCGTGACTGGAGTTC	ACACTC		
DS07	CAAGCAGAAGACGGCATACGAGATACAGACGTGACTGGAGTTC	ACAGAC		
DS08	CAAGCAGAAGACGGCATACGAGATACAGTGGTGACTGGAGTTC	ACAGTG		
DS09	CAAGCAGAAGACGGCATACGAGATACCACTGTGACTGGAGTTC	ACCACT		
DS10	CAAGCAGAAGACGGCATACGAGATACCAGAGTGACTGGAGTTC	ACCAGA		
DS11	CAAGCAGAAGACGGCATACGAGATACCTCAGTGACTGGAGTTC	ACCTCA		
DS12	CAAGCAGAAGACGGCATACGAGATACCTGTGTGACTGGAGTTC	ACCTGT		
DS13	CAAGCAGAAGACGGCATACGAGATACGACAGTGACTGGAGTTC	ACGACA		
DS14	CAAGCAGAAGACGGCATACGAGATACGAGTGTGACTGGAGTTC	ACGAGT		
DS15	CAAGCAGAAGACGGCATACGAGATACGTCTGTGACTGGAGTTC	ACGTCT		
DS16	CAAGCAGAAGACGGCATACGAGATACGTGAGTGACTGGAGTTC	ACGTGA		
DS17	CAAGCAGAAGACGGCATACGAGATACTCACGTGACTGGAGTTC	ACTCAC		
DS18	CAAGCAGAAGACGGCATACGAGATACTCTGGTGACTGGAGTTC	ACTCTG		
DS19	CAAGCAGAAGACGGCATACGAGATACTGAGGTGACTGGAGTTC	ACTGAG		
DS20	CAAGCAGAAGACGGCATACGAGATACTGTCGTGACTGGAGTTC	ACTGTC		
DS21	CAAGCAGAAGACGGCATACGAGATAGACACGTGACTGGAGTTC	AGACAC		
DS22	CAAGCAGAAGACGGCATACGAGATAGACTGGTGACTGGAGTTC	AGACTG		
DS23	CAAGCAGAAGACGGCATACGAGATAGAGAGGTGACTGGAGTTC	AGAGAG		
DS24	CAAGCAGAAGACGGCATACGAGATAGAGTCGTGACTGGAGTTC	AGAGTC		
DS25	CAAGCAGAAGACGGCATACGAGATAGCACAGTGACTGGAGTTC	AGCACA		
DS26	CAAGCAGAAGACGGCATACGAGATAGCAGTGTGACTGGAGTTC	AGCAGT		
DS27	CAAGCAGAAGACGGCATACGAGATAGCTCTGTGACTGGAGTTC	AGCTCT		
DS28	CAAGCAGAAGACGGCATACGAGATAGCTGAGTGACTGGAGTTC	AGCTGA		
DS29	CAAGCAGAAGACGGCATACGAGATAGGACTGTGACTGGAGTTC	AGGACT		
DS30	CAAGCAGAAGACGGCATACGAGATAGGAGAGTGACTGGAGTTC	AGGAGA		
DS31	CAAGCAGAAGACGGCATACGAGATAGGTCAGTGACTGGAGTTC	AGGTCA		
DS32	CAAGCAGAAGACGGCATACGAGATAGGTGTGTGACTGGAGTTC	AGGTGT		
DS33	CAAGCAGAAGACGGCATACGAGATAGTCAGGTGACTGGAGTTC	AGTCAG		
DS34	CAAGCAGAAGACGGCATACGAGATAGTCTCGTGACTGGAGTTC	AGTCTC		
DS35	CAAGCAGAAGACGGCATACGAGATAGTGACGTGACTGGAGTTC	AGTGAC		
DS36	CAAGCAGAAGACGGCATACGAGATAGTGTGGTGACTGGAGTTC	AGTGTG		
DS37	CAAGCAGAAGACGGCATACGAGATATCCGGGTGACTGGAGTTC	ATCCGG		
DS38	CAAGCAGAAGACGGCATACGAGATATCGCGGTGACTGGAGTTC	ATCGCG		
DS39	CAAGCAGAAGACGGCATACGAGATATCGGCGTGACTGGAGTTC	ATCGGC		
DS40	CAAGCAGAAGACGGCATACGAGATATGCCGGTGACTGGAGTTC	ATGCCG		
DS41	CAAGCAGAAGACGGCATACGAGATATGCGCGTGACTGGAGTTC	ATGCGC		
DS42	CAAGCAGAAGACGGCATACGAGATATGGCCGTGACTGGAGTTC	ATGGCC		
DS43	CAAGCAGAAGACGGCATACGAGATCAACCTGTGACTGGAGTTC	CAACCT		
DS44	CAAGCAGAAGACGGCATACGAGATCAACGAGTGACTGGAGTTC	CAACGA		
DS45	CAAGCAGAAGACGGCATACGAGATCAAGCAGTGACTGGAGTTC	CAAGCA		
DS46	CAAGCAGAAGACGGCATACGAGATCAAGGTGTGACTGGAGTTC	CAAGGT		
DS47	CAAGCAGAAGACGGCATACGAGATCACAAGGTGACTGGAGTTC	CACAAG		
DS48	CAAGCAGAAGACGGCATACGAGATCACATCGTGACTGGAGTTC	CACATC		
DS49	CAAGCAGAAGACGGCATACGAGATCACTACGTGACTGGAGTTC	CACTAC		
DS50	CAAGCAGAAGACGGCATACGAGATCACTTGGTGACTGGAGTTC	CACTTG		
DS51	CAAGCAGAAGACGGCATACGAGATCAGAACGTGACTGGAGTTC	CAGAAC		
DS52	CAAGCAGAAGACGGCATACGAGATCAGATGGTGACTGGAGTTC	CAGATG		
DS53	CAAGCAGAAGACGGCATACGAGATCAGTAGGTGACTGGAGTTC	CAGTAG		
DS54	CAAGCAGAAGACGGCATACGAGATCAGTTCGTGACTGGAGTTC	CAGTTC		
DS55	CAAGCAGAAGACGGCATACGAGATCATCCAGTGACTGGAGTTC	CATCCA		
DS56	CAAGCAGAAGACGGCATACGAGATCATCGTGTGACTGGAGTTC	CATCGT		
DS57	CAAGCAGAAGACGGCATACGAGATCATGCTGTGACTGGAGTTC	CATGCT		
DS58	CAAGCAGAAGACGGCATACGAGATCATGGAGTGACTGGAGTTC	CATGGA		
DS59	CAAGCAGAAGACGGCATACGAGATCCAACGGTGACTGGAGTTC	CCAACG		
DS60	CAAGCAGAAGACGGCATACGAGATCCAAGCGTGACTGGAGTTC	CCAAGC		
DS61	CAAGCAGAAGACGGCATACGAGATCCATCCGTGACTGGAGTTC	CCATCC		
DS62	CAAGCAGAAGACGGCATACGAGATCCATGGGTGACTGGAGTTC	CCATGG		
DS63	CAAGCAGAAGACGGCATACGAGATCCGCAAGTGACTGGAGTTC	CCGCAA		
DS64	CAAGCAGAAGACGGCATACGAGATCCGCTTGTGACTGGAGTTC	CCGCTT		
DS65	CAAGCAGAAGACGGCATACGAGATCCGGATGTGACTGGAGTTC	CCGGAT		
DS66	CAAGCAGAAGACGGCATACGAGATCCGGTAGTGACTGGAGTTC	CCGGTA		
DS67	CAAGCAGAAGACGGCATACGAGATCCTACCGTGACTGGAGTTC	CCTACC		
DS68	CAAGCAGAAGACGGCATACGAGATCCTAGGGTGACTGGAGTTC	CCTAGG		
DS69	CAAGCAGAAGACGGCATACGAGATCCTTCGGTGACTGGAGTTC	CCTTCG		
DS70	CAAGCAGAAGACGGCATACGAGATCCTTGCGTGACTGGAGTTC	CCTTGC		
DS71	CAAGCAGAAGACGGCATACGAGATCGAACCGTGACTGGAGTTC	CGAACC		
DS72	CAAGCAGAAGACGGCATACGAGATCGAAGGGTGACTGGAGTTC	CGAAGG		
DS73	CAAGCAGAAGACGGCATACGAGATCGATCGGTGACTGGAGTTC	CGATCG		
DS74	CAAGCAGAAGACGGCATACGAGATCGATGCGTGACTGGAGTTC	CGATGC		
DS75	CAAGCAGAAGACGGCATACGAGATCGCCAAGTGACTGGAGTTC	CGCCAA		
DS76	CAAGCAGAAGACGGCATACGAGATCGCCTTGTGACTGGAGTTC	CGCCTT		
DS77	CAAGCAGAAGACGGCATACGAGATCGCGATGTGACTGGAGTTC	CGCGAT		
DS78	CAAGCAGAAGACGGCATACGAGATCGCGTAGTGACTGGAGTTC	CGCGTA		
DS79	CAAGCAGAAGACGGCATACGAGATCGGCATGTGACTGGAGTTC	CGGCAT		
DS80	CAAGCAGAAGACGGCATACGAGATCGGCTAGTGACTGGAGTTC	CGGCTA		
DS81	CAAGCAGAAGACGGCATACGAGATCGTACGGTGACTGGAGTTC	CGTACG		
DS82	CAAGCAGAAGACGGCATACGAGATCGTAGCGTGACTGGAGTTC	CGTAGC		
DS83	CAAGCAGAAGACGGCATACGAGATCGTTCCGTGACTGGAGTTC	CGTTCC		
DS84	CAAGCAGAAGACGGCATACGAGATCGTTGGGTGACTGGAGTTC	CGTTGG		
DS85	CAAGCAGAAGACGGCATACGAGATCTACCAGTGACTGGAGTTC	CTACCA		
DS86	CAAGCAGAAGACGGCATACGAGATCTACGTGTGACTGGAGTTC	CTACGT		
DS87	CAAGCAGAAGACGGCATACGAGATCTAGCTGTGACTGGAGTTC	CTAGCT		
DS88	CAAGCAGAAGACGGCATACGAGATCTAGGAGTGACTGGAGTTC	CTAGGA		
DS89	CAAGCAGAAGACGGCATACGAGATCTCAACGTGACTGGAGTTC	CTCAAC		
DS90	CAAGCAGAAGACGGCATACGAGATCTCATGGTGACTGGAGTTC	CTCATG		

DS91	CAAGCAGAAGACGGCATACGAGATCTCTAGGTGACTGGAGTTC	CTCTAG
DS92	CAAGCAGAAGACGGCATACGAGATCTCTTCGTGACTGGAGTTC	CTCTTC
DS93	CAAGCAGAAGACGGCATACGAGATCTGAAGGTGACTGGAGTTC	CTGAAG
DS94	CAAGCAGAAGACGGCATACGAGATCTGATCGTGACTGGAGTTC	CTGATC
DS95	CAAGCAGAAGACGGCATACGAGATCTGTACGTGACTGGAGTTC	CTGTAC
DS96	CAAGCAGAAGACGGCATACGAGATCTGTTGGTGACTGGAGTTC	CTGTTG
Barcode primer R	AATGATACGGCGACCACCGAGATCTACACTCTTTCCCTACACGAC	

Supplemental Table 3

Primer	Mean Length (range)	Kingdom	Phylum	Class	Order	Family	Genus	Species	# Unique sequences
16SArchaea	152 bp (51 - 288)	1.00 (1.00)	1.00 (1.14)	1.02 (1.14)	1.02 (1.14)	1.17 (1.22)	3.13 (1.84)	3.60 (8.73)	3029
16Smam	95 bp (76 - 118)	1.00 (1.01)	1.03 (1.01)	1.03 (1.01)	1.00 (1.01)	1.03 (1.01)	1.06 (1.10)	1.27 (1.46)	727
23SrDNA	334 bp (65 - 341)	1.00 (1.01)	1.00 (1.02)	1.07 (1.04)	1.06 (1.31)	1.14 (1.57)	1.25 (2.06)	1.34 (3.64)	2944
AmpCB	68 bp (68 - 68)	1.00 (1.00)	1.00 (1.00)	1.00 (1.00)	1.00 (1.00)	1.00 (1.00)	1.00 (1.01)	1.00 (1.14)	20
Aves12S	53 bp (50 - 211)	1.00 (1.00)	1.00 (1.00)	1.00 (1.00)	1.01 (1.01)	1.41 (1.05)	2.99 (1.26)	12.39 (1.57)	134
BryoTrnL	52 bp (25 - 55)	1.00 (1.00)	1.01 (1.00)	1.01 (1.00)	1.03 (1.05)	2.13 (1.39)	9.78 (2.12)	20.07 (3.74)	262
COI_ZBJ_Art	162 bp (156 - 166)	1.00 (1.00)	1.00 (1.00)	1.00 (1.00)	1.00 (1.00)	1.15 (1.05)	1.23 (1.09)	1.27 (1.18)	26
Cop28S	209 bp (53 - 368)	1.00 (1.00)	1.00 (1.00)	1.00 (1.00)	1.00 (1.00)	1.00 (1.01)	1.00 (1.03)	2.04 (1.14)	50
Diatom18S	359 bp (52 - 424)	1.00 (1.00)	1.50 (1.00)	1.02 (1.01)	1.10 (1.04)	1.21 (1.05)	1.38 (1.09)	1.62 (1.55)	1882
FishCB	90 bp (76 - 192)	1.00 (1.00)	1.00 (1.00)	1.00 (1.00)	1.00 (1.01)	1.00 (1.01)	1.01 (1.08)	1.45 (1.29)	229
FungusITS	163 bp (73 - 294)	1.01 (1.01)	1.03 (1.08)	1.05 (1.10)	1.07 (1.11)	1.07 (1.12)	1.60 (1.26)	2.08 (2.64)	1527
trnL	48 bp (17 - 80)	1.00 (1.00)	1.01 (1.00)	1.04 (1.00)	1.13 (1.01)	1.21 (1.01)	9.34 (4.05)	34.89 (14.33)	3113

Supplemental Table 5

**Species**	**Fish mt-Cytb**	**Mammalian 16S rRNA**
Central stoneroller		✓
White sucker		✓
Grass/Black carp		✓
Cyprinella spiloptera		✓
Common carp	✓	✓
Cypress minnow		✓
Northern hogsucker		✓
Shiner		✓
Spotted sucker		✓
Redhorse		✓
Bluehead chub		✓
Emerald shiner		✓
Blacknose dace		✓
Bluntnose minnow		✓
Creek chub	✓	

Supplemental Table 7

Extraction (DNeasy 91 samples)	$440.00
First PCRs (12 primers)	$335.00
Second PCR	$28.00
Sequencing (MiSeq 150 PE)	$1,200.00
**Total (91 samples)**	**$2,003.00**
**Per sample**	**$22.01**
